# ﻿A recircumscription of *Geocharis* (Zingiberaceae) as a result of the discovery of a new species in Sumatra, Indonesia

**DOI:** 10.3897/phytokeys.244.119306

**Published:** 2024-07-01

**Authors:** Nurainas Nurainas, Witri Zulaspita, Thoriq Alfath Febriamansyah, Syamsuardi Syamsuardi, Axel Dalberg Poulsen

**Affiliations:** 1 Herbarium Universitas Andalas (ANDA), Biology Department, Faculty of Mathematics and Natural Sciences, Universitas of Andalas, Limau Manis, Padang 25163, West Sumatra, Indonesia; 2 Biology Department, Faculty of Mathematics and Natural Sciences, University of Andalas, Padang 25163, Indonesia; 3 Royal Botanic Garden Edinburgh, Science, 20A Inverleith Row, Edinburgh EH3 5LR, Scotland, UK

**Keywords:** *Geocharisglobosa*, globose fruit, Mount Marapi, taxonomy, West Sumatra, wild gingers

## Abstract

Recent fieldwork conducted in Sumatra resulted in unusual collections of the conspicuous ginger genus *Geocharis*, a genus that harbours a total of six species distributed in Sumatra, the Malay Peninsula, Borneo and the Philippines. After carefully reviewing types and protologues of existing taxa, we conclude that the recent collections represent a new species described here as *Geocharisglobosa*, which is similar to *G.aurantiaca*, *G.rubra* and *G.radicalis* by the flowers spreading evenly in all directions but differs from these by the less divided labellum and by the unstructured and smooth globose fruits. We provide a detailed description and a photographic plate as well as a preliminary Red List Assessment for the new species. With the new species added to *Geocharismacrostemon* and *G.radicalis*, the number of species in Sumatra hereby increases to three. A recircumscription of the genus is provided, taking into account the unusual fruit character of the new species.

## ﻿Introduction

**Figure 1.**
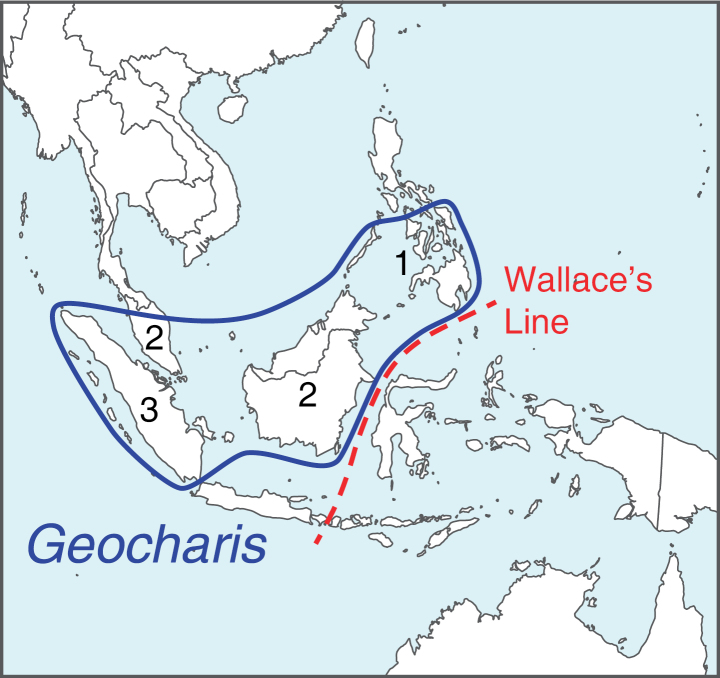
Distribution map of *Geocharis*. Number of species indicated for Sumatra including *G.globosa* described in the present paper. Map by A.D. Poulsen.

The ginger genus *Geocharis* (K.Schum.) Ridl. (Ridley 1908) harbours only eight taxa: six species, two of which have two varieties each (World Flora Online Plant List 2023; Table 1) distributed in Sumatra, the Malay Peninsula, Borneo, and the Philippines (Newman et al. 2004; Fig. 1). Morphologically, this genus is characterized by having a radical inflorescence with flowers borne in cincinni of up to two flowers and it may easily be distinguished from other genera in the Zingiberaceae by the tessellate pattern (markings in squares and rectangles) on the leaf sheath, a linear and divided labellum and a broad, toothed filament formed by fusion with the lateral staminodes. Sampling three species of the genus (Geocharisfusiformis(Ridl.)R.M.Sm.var.borneensis R.M.Sm, *G.macrostemon* (K.Schum.) Holttum, *G.rubra* Ridl.) in a molecular-based study, Poulsen et al. (2018), demonstrated that the genus is monophyletic and sister to the genus *Sulettaria* A.D.Poulsen & Mathisen.

**Table 1.** Accepted taxa of *Geocharis* and their distribution.

Species	Distribution
*G.aurantiaca* Ridl.	Peninsula Malaysia
G.fusiformis(Ridl.)R.M.Sm.var.fusiformis	Philippines
G.fusiformisvar.borneensis R.M.Sm.	Borneo
*G.macrostemon* (K.Schum.) Holttum	Sumatra
*G.radicalis* (Valeton) B.L.Burtt & R.M.Sm.	Sumatra
G.rubraRidl.varrubra	Borneo
G.rubravar.puberula Meekiong	Borneo
*G.secundiflora* (Ridl.) Holttum	Peninsular Malaysia

Two species of *Geocharis* occur in Sumatra, *G.macrostemon* and *G.radicalis* (Valeton) B.L.Burtt & R.M.Sm., of which only a few collections have been made (Holttum 1950; Newman et al. 2004). *Geocharismacrostemon* was first collected in 1878 by the Italian naturalist, Odoardo Beccari, in West Sumatra, in the lowland forest at Ajer Mantjoer (Lembah Anai). Currently, this is a conservation area within the Anai Valley Nature Reserve. Despite several surveys of the reserve, we were unable to collect this species again.

*Geocharisradicalis* was collected by Lörzing (*Lörzing 5912*) in the forest at 1000 m elevation near Deli, North Sumatra. Valeton (1921) first placed this species in *Rhynchanthus* Hook.f. whereas Burtt and Smith combined it in *Geocharis* in 1972. New material of this species is lacking and desirable.

During our fieldwork conducted in Sumatra since 2020, collections of *Geocharis* were made at Mount Marapi, West Sumatra, which differed from the two known Sumatran species mentioned above. Comparisons made to all known species of the genus, also did not result in a match.

## ﻿Materials and methods

We examined collections deposited at several herbaria (ANDA, BO, E, FI, K, L, KEP, SAN, SING), as well as and high-resolution photographs of specimens (herbarium abbreviations follow Thiers 2022). A literature review was conducted on other species of *Geocharis* (Holttum 1950; Smith 1986; POWO 2023).

Morphological descriptions were primarily obtained from living material collected in the field. Fertile material was preserved in spirit while other parts were dried and pressed for the herbaria. Detail characters were observed using a stereomicroscope. The measurements were made using a ruler and image-J software. The distribution map was based on existing records as a guideline. Terminology follows Stearn (1983), Harris and Harris (2001) and Beentje (2010).

## ﻿Results

The recently collected material is clearly a species of *Geocharis* due to the distinct pattern of the leaf sheath, the narrow and incised labellum and the broad and toothed filament formed by fusion with the lateral staminodes. The smooth, glabrous and globose fruits are unlike any known species and below we update a circumscription of the genus as well as describe the new species.

### ﻿Taxonomic treatment

#### ﻿ Geocharis

Taxon classificationPlantaeZingiberalesZingiberaceae

(K.Schum.) Ridl.

896641FF-661E-5E42-AA00-60B8737FDE84

Alpinia Sect. Geocharis K.Schum., Pflanzenr. IV, 46 (1904) 363.

##### Type.

*Geocharismacrostemon* (K.Schum.) Holttum (designated by De Boer et al. 2018: 31).

##### Description.

Terrestrial herb with creeping rhizome, rhizome scales tessellate (with markings in squares) similar to the sheath. The leafy shoot of medium height. Sheath with remarkable white-felted cross-bars between the longitudinal ribs. Flowering shoots radical; peduncle long-prostrate, ascending or erect, with cross-barred sheaths, rachis ± curved with many flowers spreading evenly in all directions or secund. Inflorescence lax, bracts never imbricate, ± soon falling off, subtending at least two flowers in the lowermost bracts. Bracteole tubular at least at the very base. Flowers orange to red, shortly pedicellate. Calyx tubular, trilobed, apices toothed. Floral tube as long as calyx or longer. Dorsal corolla lobe larger than the laterals, enclosing the filament. Staminal tube (formed by fusion of labellum and filament) present above insertion of corolla lobes. Labellum narrow, deeply bilobed, incised in upper half or to base, lobes ± linear. Staminodes fused with filament forming a semi-tube broader than anther, apices tooth-like just below the anther. Anther oblong, crest entire, cucullate; thecae pa­rallel, dehiscing through their entire length. Ovary globose to ellipsoid. Epigynous gland short. Stigma obcuneate. Fruit globose to elongate, smooth or ridged, often warty, glabrous or pubescent, base of calyx persistent. Seeds angular, arillate.

##### Etymology.

The name means ground (geo) beauty (charis).

##### Distribution.

The genus *Geocharis* occurs in Sumatra, Peninsular Malaysia, Sarawak, Sabah, and the Philippines (Fig. 1).

##### Note.

Holttum (1950) and Smith (1986) made circumscriptions of *Geocharis*, which we have considered and expanded, taking into account the new material resulting in the new species described below.

#### ﻿ Geocharis globosa

Taxon classificationPlantaeZingiberalesZingiberaceae

Zulaspita & Nurainas sp. nov.

3947D5E2-9E4B-5350-822C-23185025DD6D

urn:lsid:ipni.org:names:77344563-1

Fig. 2

##### Diagnosis.

The new species is similar to *G.aurantiaca*, *G.rubra* and *G.radicalis* by the flowers spreading in all directions from an erect flowering shoot but differs from all of these by the labellum being incised only halfway from apex (not deeply or completely divided), and having a smooth and glabrous surface to the ovary and globose fruits (Table 2).

**Table 2.** A comparison of species of *Geocharis* with an erect flowering shoot and flowers pointing in all directions (not secund).

Character	*G.aurantiaca*	*G.globosa*	*G.radicalis*	*G.rubra*
Labellum incision	Deeply bilobed	Halfway	Split to base	Deeply bilobed
Fruit shape	Ellipsoid	Globose (to ellipsoid)	–	Ellipsoid
Fruit macrostructure	–	Smooth	–	Ridged and grooved
Fruit surface	Verrucose	Smooth	Verrucose	Rugose

**Figure 2.**
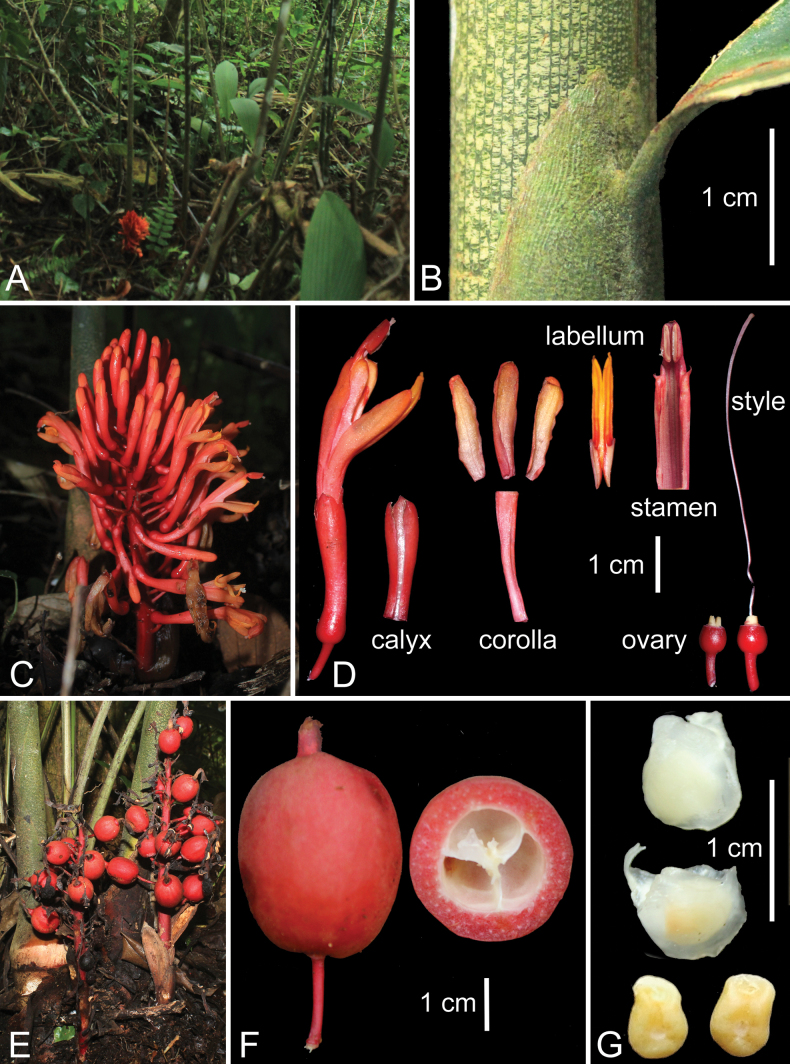
*Geocharisglobosa* Zulaspita & Nurainas, sp. nov. **A** habit **B** sheath (with tessellate pattern), ligule and base of leaf blade **C** inflorescence **D** flower dissection **E** infructescence **F** fruit **G** seeds with and without aril. Photographs by T.A. Febriamansyah, edited by A.D. Poulsen.

##### Type.

Indonesia. West Sumatera province, Tanah Datar District, Andaleh village, 0°26'38.96"N, 100°27'20.41"E, 1149 m elevation, 26 March 2022, *Witri Zulaspita et al.* WZ29 (Holotype ANDA; isotype SING).

##### Description.

Terrestrial, evergreen herb, forming clumps of 3–5 leafy shoots. Rhizome subterranean, 0.5–1.0 cm diam., aromatic; scales triangular, 4–6 × 2.5–3 cm, longitudinally ribbed, pale red with whitish green when fresh, pale brown when dry, lanate. Leafy shoots 2–2.5 m tall, 8–21 leaves per shoot, 13–15 cm apart; base 3.0–3.5 cm diam., bright pink; sheath dark green with irregular small horizontal white bars joining the longitudinal ribs, pubescent; ligule shortly bilobed, to 1.5 cm long, dark green, tomentose; petiole canaliculate, 1.0–1.5 cm long, green, glabrous; lamina narrowly ovate to oblong-elliptic, 48–58 × 9–10 cm, green adaxially and pale green abaxially, longitudinal ribbed, puberulent above, pubescent abaxially at margin, base attenuate, margin entire, apex caudate (1.5–2 cm long). Flowering shoot arising from rhizome, 10–14 cm distance from the base of the leafy shoot, ascending to erect, 10–25 cm long with 10–45 flowers distributed evenly in all directions, 5–15 flowers open at a time, rachis 7–15 long; peduncle 3–8 × 0.5 cm, red, glabrous; peduncular bract broadly ovate, 1.5–3 × 1–1.5 cm, pale red with irregular small horizontal white bars joining the longitudinal ribs, apex subapically mucronate; floral bract soon falling off, narrowly elliptic, 5–9.5 × 2.0–3.5 cm, reddish-brown, glabrous, subapically mucronate. Bracteole narrowly elliptic, slit to base on one side, 5–7 mm long, transparent, caducous, apex irregularly bilobed, glabrous. Flower slender, 5–6 cm long; flowering pedicel 0.5–2 cm long, red, glabrous; calyx tubular, 2.0–2.5 × 0.5 cm, trilobed, puberulent, red; corolla tube 2.0–2.5 × 0.3 cm, bright pink, hirsute; dorsal corolla lobe oblong-elliptic, 1.5–2.0 × 0.5 cm, apex rounded, yellowish to red, glabrous, lateral corolla lobes oblong-elliptic, 1.5 cm × 0.3 cm, apex rounded, yellowish-red, glabrous; staminal tube ca. 5 mm long; labellum linear, bilobed, split half from apex, 10–15 × 4–5 mm, crimson with yellow edges, apex obtuse, glabrous; stamen 23–28 mm, reddish yellow, glabrous; filament 18–22 × 6–10 mm (incl. fusion with staminodes) free part 2–3 mm long, flattened, lateral staminodes tooth-like, ca. 2 mm long, pale violet, glabrous; anther 5–6 × 3–4 mm, white, puberulent, thecae, dehiscent through their entire length, pubescent; anther crest rounded, c. 1 mm long, slightly trilobed, dark-red, glabrous; ovary globose, 7 × 7 mm, smooth, red, puberulent; epigynous gland bipartite, rounded, 2 × 2 mm, yellowish, glabrous; style c. 5.5 cm long, pinkish white, setose; stigma clavate (with a lateral knob), pinkish white, ostiole apical, semi-circular, 0.5 mm across, margin ciliate. Fruit globose to slightly ellipsoid, 2–4 × 2–3 cm, reddish-orange, glabrous. Seeds irregular, ca. 6 × 5 mm (immature), aril white.

##### Distribution and habitat.

Endemic to Sumatra and known only from the type locality (Mt. Marapi, West Sumatra). Montane forest, moist forest understorey.

##### Phenology.

Flowering in March to June and fruiting in July to September.

##### Etymology.

The epithet refers to the shape of the fruit.

##### Conservation status and preliminary IUCN Red List assessment.

*G.globosa* is only known from the type locality of Mt Marapi, Andaleh, West Sumatra. The population of *G.globosa* has so far only been found at Andaleh in the border zone between Mount Merapi Nature Park and local agricultural areas. Following the IUCN Standards and Petitions Committee (2022), *G.globosa* we assess here preliminarily the status as Critically endangered (CR) based on EOO <100 km^2^ (B1), limited geographic range (B1+ a, b) and a population of less than 20 individuals (D).

##### Additional specimens examined (paratypes).

Indonesia. Sumatra. West Sumatra province, Tanah Datar District, Andaleh village, 0.442992756°S, 100.4552318°E, 1149 m elev., 2 July 2022. fruiting, *W. Zulaspita et al. WZ30* (ANDA); Tanah Datar District, Andaleh village, 0.442119833°S, 100.4542314°E, 1149 m elev, 2 July 2022, fruiting, *W. Zulaspita et al. WZ31* (ANDA); Tanah Datar District, Andaleh village, 0.440810449°S, 100.4549408°E, 1149 m elev., 30 July 2022, fruiting, *W. Zulaspita et al. WZ32* (ANDA); Tanah Datar District, Singgalang village, Trails to Mount Tandikek, 0.449753522°S, 100.3396321°E, 1195 m elev., 24 September 2023, flowering and fruiting.

##### Notes.

*Geocharisglobosa* is easily recognized by the completely smooth fruits, which are unlike any other known species of the genus. In Sumatra, it is most similar with *G.radicalis* that also has an erect flowering shoot with flowers pointing in all directions.

Further studies are needed to evaluate the usefulness of any vegetative characters, especially the ligule and petiole. The importance of pickled flowers and fruits must again be emphasized as previously pointed out by Khaw (2001) and Poulsen (2006). With only limited information about the detailed flower structure and generally lacking pickled flowers, there is yet much to learn about the floral morphology and its variation in *Geocharis*.

## Supplementary Material

XML Treatment for Geocharis

XML Treatment for Geocharis globosa

## ﻿Additional information

### Author ORCIDs

Nurainas Nurainas https://orcid.org/0000-0003-1682-2976

Witri Zulaspita https://orcid.org/0000-0003-0820-9872

Thoriq Alfath Febriamansyah https://orcid.org/0009-0004-6684-9573

Syamsuardi Syamsuardi https://orcid.org/0000-0001-8351-6528

Axel Dalberg Poulsen https://orcid.org/0000-0002-7651-6439

### Data availability

All of the data that support the findings of this study are available in the main text.
